# Sub-Acute Syndesmotic Injury: A Review and Proposed Treatment Algorithm

**DOI:** 10.7759/cureus.16670

**Published:** 2021-07-27

**Authors:** Urpinder S Grewal, Crispin Southgate, Baljinder S Dhinsa

**Affiliations:** 1 Trauma and Orthopaedics, East Kent NHS Trust, Ashford, GBR

**Keywords:** sub-acute, syndesmotic, injury, treatment, algorithm, syndesmosis

## Abstract

Sub-acute syndesmotic injuries are classified as from six weeks to six months from the initial injury date and can be considered a distinct group of patients; however, they are often mistreated and progress to chronic injuries with significant sequelae.

The authors performed a comprehensive literature search on the MEDLINE database. The search yielded 165 studies up to January 2021, after the application of inclusion/exclusion criteria. This yielded 10 studies with a total of 156 relevant patients for review.

We found that a delay in diagnosis is common and has a negative impact on outcomes. If a sub-acute syndesmotic injury is suspected and plain radiographs are inconclusive, magnetic resonance imaging is indicated if there is still an index of suspicion. Surgical intervention should aim to restore normal length and rotational alignment of the fibula whilst also addressing the need to debride tissues within the joint and syndesmosis. Syndesmosis must then be adequately reduced and stabilised with syndesmotic screw fixation, and augmentation with tendon/ligament reconstruction should be considered. All studies showed an average improvement in functional outcome measures post-operatively. The only study to compare sub-acute and chronic patients’ functional outcomes post-operatively showed significant improvement in the sub-acute cohort; highlighting the importance of early intervention.

We suggest a treatment algorithm that may help with the diagnosis and management of these injuries. We believe this will help all healthcare professionals to standardise care. Further research is required to assess sub-acute injury outcomes with tendon/ligamentous augmented reconstruction, as no level 1 or 2 studies currently exist.

## Introduction and background

Introduction

The management of acute syndesmotic injury has a clear consensus, and the number of treatment algorithms for chronic syndesmotic injury has also expanded over the last decade. However, a new subgroup has recently been classified. The European Society of Sports Traumatology Knee Surgery and Arthroscopy-Ankle & Foot Associates (ESSKA-AFAS) consensus panel recently subclassified syndesmotic injury into three groups according to the time frame from index injury. These are as follows, acute (less than six weeks), sub-acute (six weeks to six months) and chronic (more than six months) [1].

Management of sub-acute syndesmotic injuries is critical to prevent the onset of degenerative change associated with poor functional outcomes and patient morbidity. Therefore, this subgroup is critical, as timely and proper intervention is required. Prompt diagnosis of syndesmotic injuries and reduction in the number of acutely mismanaged cases would lead to a reduction in the number of cases presenting in the sub-acute category. However, some cases will continue to fall into the sub-acute category, development of algorithms such as the one presented here will aim to reduce further patient morbidity.

This article presents an algorithmic approach to the treatment of sub-acute syndesmotic injuries based on evidence in recent studies for both sub-acute and chronic syndesmotic injuries. Chronic injury studies were only included if patients underwent reconstructive procedures in the absence of degenerative changes. We will also outline clinical tests and imaging techniques for such injuries; with the aim of helping clinicians make a prompt clear diagnosis.

Sub-acute syndesmotic injuries often present with non-specific symptoms and radiographic findings. It can often present as either a result of mal-reduction of a fracture or missed diagnosis. Left untreated significant functional impairment and pain may result, hence prompt diagnosis is important if the patient is to have an optimum outcome.

It is widely recognized that a missed or untreated syndesmotic injury and its resultant instability leads to long-term functional impairment and pain. A case series by Grass et al. noted that 20% of patients reporting to a tertiary centre for ankle arthrodesis had significant widening of the ankle mortise [2]. In addition to this, Ramsey and Hamilton described that 1 millimetre (mm) in lateral talar shift leads to a reduction in tibiotalar articulation by 42% [3]. Whilst general agreement exists regarding the management of an acute syndesmotic injury by reduction and internal fixation; currently, no such consensus exists for sub-acute syndesmotic injuries. Furthermore, in recent literature on treatment methods, this injury has often been pigeon-holed with chronic injuries.

The authors will review the available literature on this topic, with a particular focus on treatment methods and outcomes. With support from the available literature, we have provided an algorithm that we believe will help all healthcare professionals to standardise care.

Anatomy

A syndesmosis can be defined as a fibrous joint in which two adjacent bones are connected by a membrane or strong ligaments.

The distal tibiofibular syndesmotic articulation is made up of two key components, the bony and ligamentous anatomy. The bony anatomy comprises the articulation between the distal fibula convex surface and the distal tibia concave surface [4], whilst the ligamentous anatomy consists of four ligamentous structures: the anterior-inferior tibiofibular ligament (AITFL), posterior-inferior tibiofibular ligament (PITFL), interosseous ligament (IOL), and the transverse ligament [5].

The ligamentous complex stabilizes the ankle mortise by holding the fibula in the incisura fibularis of the distal tibia. Of the four ligaments, the AITFL and PITFL are considered the primary stabilizers of the distal tibiofibular articulation. The AITFL originates from the anterior tubercle of the tibia and inserts into the anterior tubercle of the distal fibula. It acts to prevent excessive translation of the fibula and prevents external rotation of the talus within the ankle mortise [6]. The PITFL originates on the posterior tubercle of the tibia and is attached to the posterior tubercle of the fibula medial to the sulcus of the lateral malleolus [7]. The IOL is a continuation of the interosseus membrane between the tibia and fibula that commences 4-5 centimetres (cm) above the level of the ankle joint line. The fibres run laterodistally from the tibia to the fibula to almost completely fill the space between the two bones [7-8]. Finally, the transverse ligament traverses at the posterior portion of the ankle joint and extends from the fibular malleolar fossa to the posteroinferior corner of the fibular notch of the distal tibia [9].

Biomechanically, the syndesmosis acts as a secondary stabilizer against talar translation [10]. Hence, unidentified syndesmotic injury can result in instability and subsequently degenerative arthritis [11].

Methodology

This was a qualitative review article based on a comprehensive literature search. We have outlined relevant information with regards to the mechanism of injury, clinical examination and management.

The aim of this article is to present an algorithmic approach to the treatment of sub-acute syndesmotic injuries based on evidence in recent studies for both sub-acute and chronic syndesmotic injuries treated with a reconstructive method. With specific regards to operative management and the proposed treatment algorithm, a detailed search methodology was undertaken and is outlined below.

Search strategy

As the sub-acute cohort of patients has only been classified since 2016, the authors made the decision to include sub-acute and chronic reconstructive studies within this review in order to increase data for review with regards to management methods.

The research was based on full-text English articles obtained using the MEDLINE database. The database was searched using the following query terms: “(((sub-acute) OR (chronic)) AND (syndesmotic or syndesmosis) OR (injury)))”. The search was conducted on January 15, 2021. The search yielded 165 reports in total. A further manual search was conducted, and one further relevant article was yielded. Therefore, a total of 166 reports were assessed. After duplicates were excluded, 116 reports remained.

Inclusion and exclusion criteria

Inclusion criteria for the study were as follows: retrospective or prospective human study investigating the results of syndesmosis reconstruction in the sub-acute or chronic setting.

Exclusion criteria were as follows: a) not published in English, b) investigating outcomes of non-reconstructive measures such as arthrodesis, c) studies greater than 20 years old, d) editorials, expert opinions and other descriptive studies, and e) cadaveric or biomechanical studies.

Study selection

**Figure 1:**
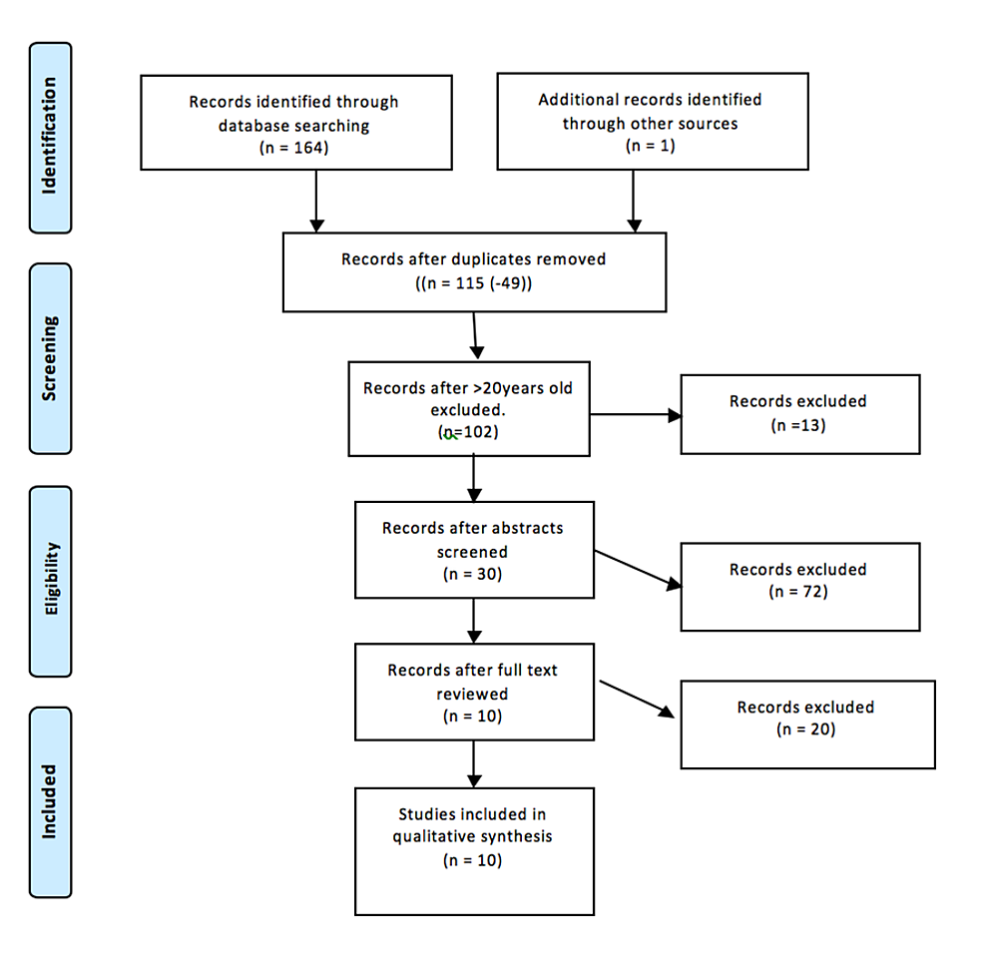
Flow diagram for study screening for sub-acute syndesmotic injury: a review and proposed treatment algorithm

After studies older than 20 years were excluded, 103 studies remained and were initially screen by title and abstract. A further 72 studies with irrelevant topics or that met the exclusion criteria were excluded. The full text of an article was then checked to ensure it met inclusion/exclusion criteria. Finally, 10 studies with a total of 156 relevant patients were included in this review. Please see Figure 1.

Quality assessment

The Coleman methodology score was used to assess the risks of bias and quality of research with regard to operative management studies [12].

Data analysis

Studies included were analysed for the number of patients included, mean follow-up duration, the mean interval between injury and surgery, post-operative functional evaluation, method of syndesmotic reconstruction and the presence of articular degeneration at the time of surgery.

## Review

Results

Mechanism of Injury

Injury to the syndesmosis may occur from an isolated ligamentous injury, fracture of the lower leg with a rotational component; or both in combination. Injury to the syndesmosis occurs in 1%-11% of all ankle sprains, whilst this percentage is felt to be higher in collision sports such as football, ice hockey and skiing [13-14].

The mechanism of injury varies in the literature; however, external rotation of the talus appears to be the most frequent causative mechanism [13]. The Lauge-Hansen classification indicates pronation-external rotation or pronation-abduction are the mechanisms most likely to lead to syndesmotic disruption [15]. Supination-external rotation mechanisms are most frequent overall and can also lead to syndesmotic disruption. Several mechanisms are therefore possible for syndesmotic injury highlighting the importance of maintaining a high level of suspicion during history taking and clinical examination.

Clinical Examination

The diagnosis and identification of an ankle syndesmosis injury can be delayed or missed due to the lack of a detailed, focused history and examination. With a thorough history taking; including mechanism of injury and focussed clinical examination most of these injuries can be identified. Appropriate imaging should then be obtained to confirm the diagnosis.

Patients who complain of pain on initiation of weight-bearing after ankle injuries should raise suspicion of syndesmotic injuries. Further suspicion should be raised when despite treatment of a prior malleolar fracture, pain continues when weight-bearing re-commences [16]. The most commonly described mechanism of injury is external rotation and dorsiflexion on a firmly planted foot [4].

Physical examination findings may include tenderness to palpation and swelling over the syndesmosis itself; in particular, the anterolateral portion [17]. Other suggestive features are limited ankle dorsiflexion; in particular, passive dorsiflexion and stiffness.

ESSKA-AFAS recommends that clinical tests include tenderness on palpation of AITFL and PITFL; the fibular translation test and the cotton test.

Palpation of syndesmotic ligaments will produce a positive test result if specific pain is elicited by direct pressure over the ligament [18]. A recent study by Sman et al. found that palpation of the syndesmotic ligaments was the most sensitive clinical test at 91% [11].

The Cotton test was initially described in 1910, and at this stage, was used to diagnose ankle fractures. It is performed by feeling the movement of the talus during translation from medial to lateral, with the ankle in a neutral position. A positive finding is documented when more movement or pain is elicited compared to the contralateral side [19].

The fibula translation test involves translating the fibula in an anteroposterior direction, whilst the tibia is held fixed. The two possible positive findings for this test are the reproduction of pain at the level of the syndesmosis during the test and increased translation in comparison to the contralateral limb [19-20].

Sman et al. also found that inability to perform a single leg hop (89%) had the highest sensitivity to diagnose a syndesmotic injury. Specificity was highest for pain out of proportion with the initial injury (79%) [21].

However, a recent systematic review found that the diagnostic sensitivity and specificity of these tests was low. It went on to suggest that if the clinical picture was suggestive of a syndesmotic injury, additional tests such as magnetic resonance imaging (MRI) and arthroscopy should be conducted [22].

Therefore, we would advocate that whilst a thorough history, examination and clinical tests are critical, no one single clinical test should be relied upon to make a final diagnosis.

Imaging

First-line diagnostic imaging remains anterior-posterior (AP), lateral and mortise radiographs. A recent systematic review found that the three most frequently used measurements to assess the syndesmosis with radiographs are the medial clear space (MCS), tibiofibular clear space (TFCS), and tibiofibular overlap [23]. Radiographs should also be used to assess for any fibula/posterior malleolus malunion, fibular shortening and the degree of degenerative change within the tibiotalar joint. The TFCS should be measured on AP and mortise radiographs one cm proximal to the distal tibia articular surface; a distance of less than six mm is considered normal. The width of the TFCS has been reported to be the most reliable radiographic indicator of syndesmotic disruption [24].

The syndesmosis is a particularly dynamic part of the ankle; hence the use of non-weight-bearing radiographs gives rise to clear radiographic inconsistencies when attempting to assess for syndesmotic disruption. The authors, therefore, welcome a recent paper by Amin et al. aiming to define tibiofibular anatomy using weight-bearing radiographs [25]. However, further research is required before these radiographic measurements can be recommended, as it is one of the first case series of its kind and does not correlate radiological findings with a clinical picture. 

Radiographs must also be taken of the full-length tibia and fibula to ensure a Maisonneuve fracture has not been missed. This fracture can occur at any location proximal to the syndesmosis, leading to rupture of the syndesmosis and instability. It is easily overlooked as in certain instances the patient may not necessarily complain of proximal fibula pain.

In the setting of non-diagnostic radiographs, two imaging modalities are frequently requested: MRI and computed tomography (CT) scans. Whilst CT may be superior to plain radiographs, multiple studies have now concluded that MRI is the investigation of choice due to its high specificity and sensitivity [26-28]. MRI is also advantageous, as it is the only non-invasive procedure allowing visualisation of AITFL and PITFL. The study by Oae et al. found a sensitivity of 100% for the diagnosis of syndesmotic disruption when compared to ankle arthroscopy findings [27]. A further series by Han et al. found MRI to be 90% sensitive and 94.8% specific in diagnosing syndesmotic injury using arthroscopic findings as a definitive diagnostic standard [26]. This series is of particular importance, as it is one of the few whose inclusion criteria selected only those with chronic syndesmotic injury. Based on these inclusion criteria, they then formulated the diagnostic criteria of MRI findings (Table 1) [26]. 

**Table 1:** Diagnostic criteria of MRI findings for chronic syndesmotic injury

MRI findings (axial and coronal images)
No visualization of syndesmosis ligament
Abnormal course, wavy, irregular contour, thickening
Increased signal in T2 and T1-weighted images

Krahenbul et al. also noted the high diagnostic accuracy by utilizing MRI specifically with regard to chronic syndesmotic instability in comparison to conventional radiographs or CT. This was felt to be due to the ability of MRI to allow direct assessment of the syndesmosis compared to CT and plain radiographs, which relied on the recognition of secondary signs [23].

Whilst CT may not be our recommended gold standard for investigation of the syndesmosis; it has a role in planning operative management of these patients. CT was noted to be particularly useful for the assessment of distal fibula and posterior malleolar malunion [23]. Therefore, CT scanning with 3D reconstruction may aid attempts at de-rotation +/- lengthening osteotomy. It can also play a role in identifying osteophytes that may need to be debrided intra-operatively [29]. We, therefore, advocate the use of CT in patients who have been identified with a sub-acute syndesmotic injury for pre-operative planning, as it highlights issues that if not addressed may prevent a satisfactory reduction of the syndesmosis.

In the setting of non-diagnostic radiographs, we would advocate that MRI is the gold standard for imaging of the syndesmosis; this is in concurrence with the ESSKA-AFAS consensus panel. CT scanning has a valuable role in pre-operative planning for the reconstruction of a sub-acute syndesmotic injury, particularly in the setting of malunited fractures

With regards to the future, it is clear that the use of static MRI for the stability of the dynamic tibiofibular joint has pitfalls. Advances in imaging techniques have led to the availability of standing bilateral CT scanning. This enables cross-sectional weight-bearing imaging of the syndesmosis. A recent study by Malhotra et al. demonstrated that weight-bearing results in lateral, posterior translation and external rotation of the fibula in relation to the incisura [30]. These dynamic changes suggest that non-weight-bearing CT imaging may be insufficient to fully evaluate a potentially injured syndesmosis. However normal reference values for the distal tibiofibular syndesmosis have only been evaluated by Patel et al. in 2019 with a sample size of 100 patients [31]. Whilst standing CT is likely to become increasingly used in the future, it cannot be recommended as the gold standard at present due to the lack of research data.

Management

Ten studies were identified outlining reconstructive management techniques for sub-acute and chronic syndesmotic injuries with a total of 156 patients. The descriptive conclusions of these studies are shown in Table 2 [10,26,32-39].

**Table 2:** Outcomes of included studies regarding sub-acute and chronic syndesmotic reconstruction

Study (year)	Injury classification	Number of included patients	Average time from injury to treatment	Method of syndesmotic reconstruction	Follow-up period (months)	Modified Coleman score	Patient outcomes
Kent et al (2020)	Sub-acute	43	Not specified	Sydndesmosis screw(s) or tightrope	Mean 49.2	41	Mean FAOS* postop pain score 94
	Chronic	29	Not specified	Syndesmotic screw(s) fixation or hamstring graft	Mean 51.6		Mean FAOS* postop pain score 83
Gross et al (2016)	Sub-acute	1	11weeks	Syndesmotic tight ropes plus medial malleolar to talus tightrope	10 (all patients followed up for this duration)	n/a	Good outcome reported; no outcome measure used
Jain et al (2014)	Sub-acute	5	10.4weeks	AITFL advancement + syndesmotic screw fixation	6 (all patients followed up for this duration)	51	Mean AOFAS** postop 88
Wagener et al (2011)	Chronic	12	24 months	AITFL advancement + syndesmotic screw fixation	Mean 25	61	Mean AOFAS post-op 92
Yasui et al (2011)	Chronic	6	12 months	Autogenous gracilis tendon using interference screws.	Mean 38	50	Mean AOFAS pre-op 53 to post-op 95
Morris et al (2009)	Chronic	8	13months	Autogenous hamstring graft using interference and syndesmosis screws	Mean 39	44	Visual analogue score pre-op 73 to postop 19. Mean AOFAS post-op 85
Zamzami et al (2009)	Chronic	11	4.7years	Autogenous semitendinous tendon plus syndesmosis screw	Mean 37.2	50	West point ankle score 95 at final follow-up
Han et al (2007)	Chronic	8	22months	Arthroscopic debridement +/- syndesmosis screw fixation	Minimum 22	59	Mean AOFAS pre-op 52 to post-op 87
Grass et al (2003)	Chronic	16	14months	Autogenous peroneus longus tendon using interference and syndesmosis screw	Mean 16.4	51	Mean Karlsson Score post-op 88. 15 out of 16 patients "relieved of pain"
Harper et al (2001)	Chronic	6	15months	Syndesmotic screw(s) fixation	Mean 24	37	Mean AOFAS pre-op 75 to post-op 91

All studies required anatomical reduction of the fibula into the incisura. Features that may prevent this are the presence of bony malunion, syndesmotic scar tissue and debris within the medial gutter. The first step is, therefore, to assess for the presence of bony malunion, such as fibula shortening, and if present, hardware must be removed and osteotomy performed [36]. Next, the syndesmosis should be debrided of scar tissue that has formed; furthermore, if scar tissue is present in the medial ankle joint gutter this should also be debrided [26,32-33,36-37,39]. Finally, if bony osteotomy was performed to achieve anatomical length and correct the rotation of the fibula, this should be held with new fixation. None of the included studies cited deltoid ligament repair as part of their reconstructive process.

The syndesmosis must now be anatomically reduced and held; four studies held this reduction with a large pointed reduction clamp [34-35,37,39]. Whilst two studies did not mention their method of holding the reduction, they stated it was done under direct vision [10,32].

Maximal stability after reduction is paramount and only two studies found syndesmotic screws alone satisfactory to maintain syndesmotic reduction [32-33]. All remaining studies reinforced reconstruction with varying methods such as autogenous tendon graft (5/10), ligamentous advancement (2/10), and, in one instance, medial malleolar to talar tight rope use. Postoperatively, seven out of 10 studies restricted patients to non-weight-bearing for a period of four to eight weeks.

With regards to patient outcomes following surgery; all studies noted mean patient-recorded outcome improvements amongst their samples. A recent study to compare outcomes of sub-acute patients alongside chronic patients noted improved FAOS of sub-acute patients in all sub-domains, including pain, symptoms, quality of life, sports and recreation, and activities of daily living, compared to those patients stabilised more than six months after injury [32]. This was the first study of its kind to compare functional outcomes of sub-acute and chronic syndesmotic injury patients.

**Figure 2:**
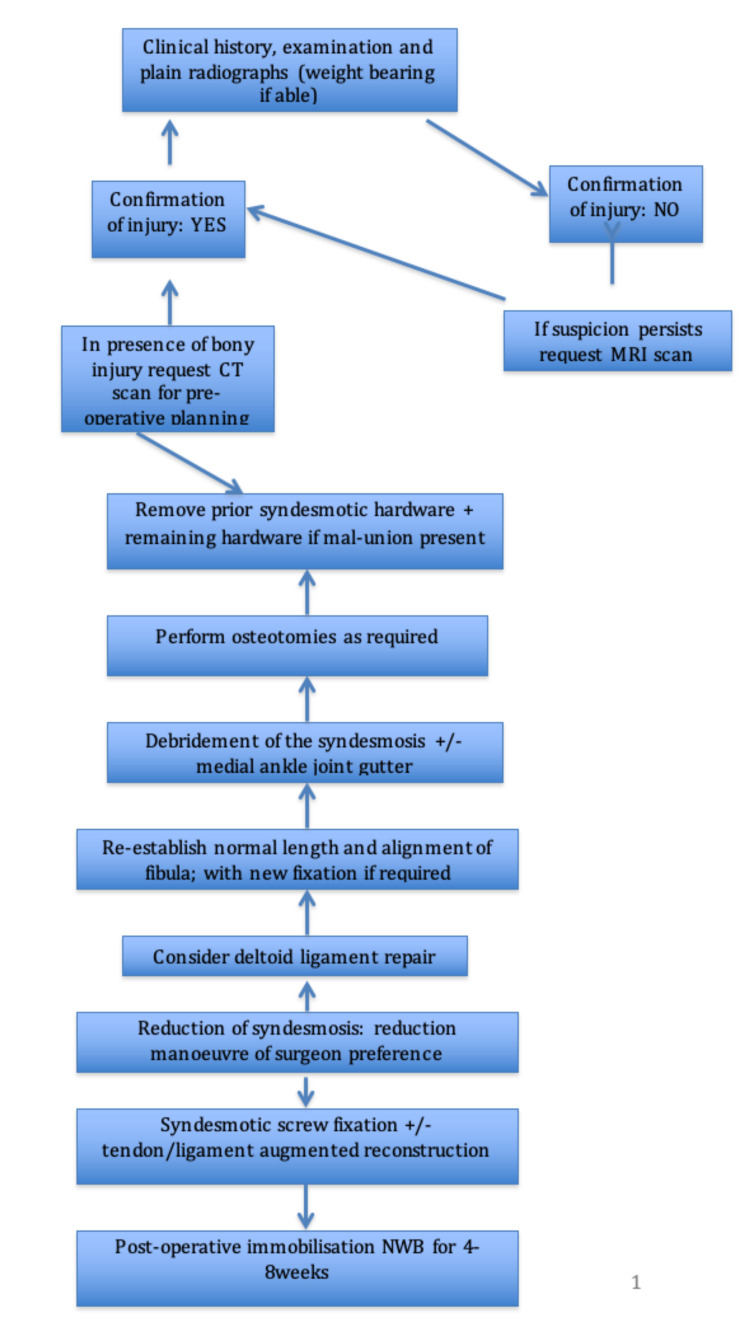
Treatment algorithm

Based on the included studies, we have formulated a management plan for sub-acute syndesmotic injuries and summarised this in the form of a treatment algorithm (Figure 2).

Discussion

Accurate diagnosis of the syndesmotic injury is the first step for the optimal treatment of a patient with a sub-acute syndesmotic injury. Plain non-weight-bearing radiographs alone will often not provide a definitive diagnosis, therefore, great care must be provided to physical examination findings and advanced imaging, such as MRI and CT scanning, must be considered.

It will be noted that treatment methods have been included in this paper in patients that are defined as in the chronic cohort. This is because reconstruction in chronic patients without osteoarthritis and sub-acute patients are similar, however, it is the number of patients with chronic syndesmotic injury and osteoarthritis that we are aiming to reduce. This paper highlights the lack of data currently unavailable with specific regards to sub-acute syndesmotic injury; with only three of 10 included studies focussing on the sub-acute patient cohort. Therefore, it is reasonable to include chronic papers that utilized reconstructive methods to discuss the surgical management of subacute injuries. In only one instance does one of these included papers describe performing a reconstructive method on a patient with marked degenerative changes, a procedure which the authors themselves describe as a failure [37]. Furthermore, as the sub-acute cohort has only recently been classified, little data currently exists, so further research will hopefully be available in future.

ESSKA clearly feel that this sub-group is important, hence its new classification, and we equally feel that its importance and identification is not currently adequate and hope our paper raises further awareness on the issue. Early recognition and management allow joint-preserving reconstructive measures; which, in turn, lead to significantly better functional outcomes. In the presence of significant degeneration, as seen in the chronic sub-cohort, arthrodesis/arthroplasty is required with an impact on function.

Sub-acute syndesmotic injuries span from ligamentous “sprains” to complex malunited fracture patterns with metalwork in situ. Despite this, reconstructive and stabilisation principles regarding syndesmosis can still follow an algorithmic approach. The accessibility of the pathway instructions enables it to be commenced by surgeons of varying experience and grade, with specific sub-speciality referral once a clear diagnosis is made. We believe that the outlined algorithm is a clear pathway in which these patients can be managed with an evidence-based approach. The algorithm is based on the literature available and no subjective comments or opinions have been added to this. Recognition and investigation of this cohort is currently poor and leads to more joint sacrificing surgery. The authors hope to reduce this impact by raising awareness and providing a protocol for investigation/management.

With regards to the management of deltoid ligament rupture in the setting of syndesmotic disruption, this remains widely controversial. The role of primary deltoid repair in the syndesmotic injury setting subgroup is not yet clearly understood, and it is noteworthy that this topic is not mentioned in any of the final studies included. Therefore, within the treatment algorithm, a deltoid ligament repair is mentioned as only a consideration based on surgeon preference.

Further research is required to assess functional outcomes of treatment for sub-acute syndesmotic injuries. An area of particular interest would be a multi-centre randomised control trial comparing syndesmotic screw fixation against newer tendon/ligament-augmented techniques with regards to both short and long-term outcomes.

Clear evidence exists to show the consequences of patients with chronic and ongoing missed syndesmotic injuries; therefore, more must be done to identify these patients in the sub-acute phase in order to optimise functional outcomes.

This paper is not presenting level 1 evidence for surgical technique. The aim of the authors is to highlight (with the limited evidence available) a significant cohort of patients that have significant functional limitations due to their injury not been identified and managed early. This is not due to negligence but a lack of understanding, and that is why we feel an algorithm is useful and can aid healthcare professionals who see such patients.

## Conclusions

A sub-acute syndesmotic injury is a significant injury that, if left untreated, increases the likelihood of a patient developing syndesmotic or tibiotalar arthritis with resultant patient morbidity. Whilst better treatment of acute injuries will lead to fewer injuries falling into this subset, mismanaged or missed injuries will persist. Therefore, treatment algorithms must exist in order to allow swift identification and standardised care, even if this involves a non-specialist clinician commencing the algorithm and referring onward. This review highlights the significant paucity of data that is lacking for a vast cohort of patients; underlying the need for more level 1 and 2 studies on their management. In the interim, we have provided a simplified treatment algorithm accessible to a range of healthcare professionals aiming to improve outcomes and raise a further spotlight on a subset of patients often pigeon-holed with non-reconstructible chronic syndesmotic injuries.
